# Allergic Contact Dermatitis in the Right Forearm Following Splint Application for Distal Radius Fracture: A Rare Case of Plaster Cotton Allergy

**DOI:** 10.7759/cureus.51802

**Published:** 2024-01-07

**Authors:** Göker Değer, Ahmet Burak Demirdas, Derya Akbaba, Muhammed Yusuf Afacan

**Affiliations:** 1 Department of Orthopaedics and Traumatology, Beykoz State Hospital, Istanbul, TUR; 2 Department of Orthopaedics and Traumatology, Istanbul University - Cerrahpasa, Cerrahpasa Faculty of Medicine, Istanbul, TUR

**Keywords:** undercast cotton padding, compartment syndrome, distal radius fracture, short arm splint, plaster of paris, allergic contact dermatitis, plaster cotton allergy

## Abstract

Allergic contact dermatitis (ACD) after splint or cast application (plaster of Paris) is infrequently encountered in orthopedic and traumatology clinical practice. This case study aims to elucidate the identification of ACD after splint application, highlight the conditions that warrant vigilance, and outline the precautions and optional treatment methods available in such instances. A 56-year-old right-hand dominant female presented to the emergency department after a fall on her right hand, manifesting pain, swelling, and tenderness without neurovascular injury. Radiographs revealed a distal radius fracture, leading to the application of a plaster of Paris splint. Within one day, she returned to the emergency department with severe itching and burning in the right arm. The splint was removed, and a dermatology consultation confirmed ACD due to undercast cotton padding. After splint removal, the patient's fracture treatment continued using a shoulder-arm sling until the lesion healed. Topical antihistamine ointment and oral corticosteroids were prescribed. Regular follow-up revealed the healing and union of the fracture by the fifth week, with minimal residual skin color changes. This case underscores the importance of prompt diagnosis and appropriate treatment in managing such occurrences. A key takeaway is the crucial need to schedule a follow-up appointment with the patient within one day of applying the cast or splint. Skin problems can emerge rather than neurovascular issues following casts or splints. Educating patients on warning signs, including skin irritation, neurovascular deficits, and symptoms of compartment syndrome, ensures the timely identification of significant issues. Healthcare practitioners should inquire about patients' histories of allergic skin reactions, taking a proactive approach to prevent ACD through early intervention and preventive measures.

## Introduction

Splint and cast applications represent the prevailing methods for stabilizing orthopedic and traumatological conditions. Instances involving fractures or dislocations of bones, soft tissue damage, and injuries to joints, tendons, strains, or sprains often necessitate stabilization through cast or splint applications. The primary objectives of these interventions are to alleviate pain and tenderness while expediting the healing process [1].

Allergic contact dermatitis (ACD) stands as a prevalent condition, with reported prevalence rates approaching 20% within the general population [2,3]. Individuals with a history of eczema are particularly susceptible to developing ACD. Sensitization to certain materials or substances may lead to pruritus, erythema, edematous skin, and, in severe cases, blistering and oozing [2]. Allergic reactions may manifest over time, with sensitivities emerging to both organic and synthetic materials. Specific allergens can be identified through patch testing, a distinctive diagnostic procedure distinguishing itself from other tests like prick, scratch, or allergy blood testing, which are incapable of diagnosing ACD. Physicians evaluate the body's reaction 48 hours post-application of patches containing specific allergens on the patient's back [2]. The management of ACD involves avoiding contact with allergens and employing antihistamine or corticosteroid treatments to mitigate allergic reactions during episodes [4].

In this context, we present the case of a 56-year-old female patient who experienced a rare allergic dermatitis resulting from undercast cotton padding following a splint application. The primary objective is to emphasize the critical importance of regular post-application checks of the casts even the splints for promptly identifying any pathological developments, as in this case, which is crucial for ensuring the ongoing health and well-being of patients undergoing orthopedic interventions.

## Case presentation

**Figure 1:**
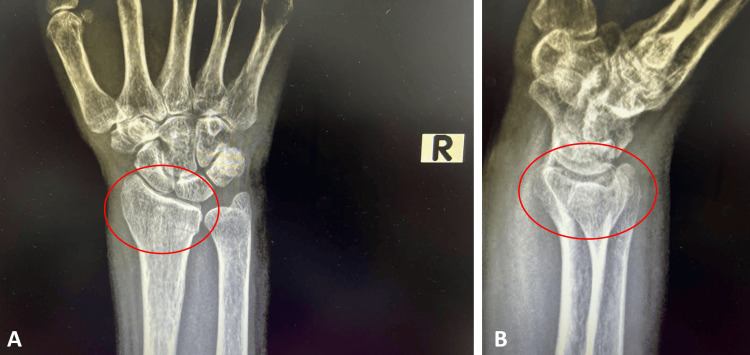
The wrist radiographs of the patient before splint application Wrist anteroposterior (1A) and lateral (1B) views of the patient prior to splint application revealed a nondisplaced fracture of the distal radius

We present an unusual case involving a 56-year-old female patient who presented to the emergency department with a severely painful and swollen right arm. The patient's medical history indicated a fall on her right hand while engaged in household cleaning. Upon physical examination, the dorsal part of the distal radius exhibited pain, tenderness, and significant swelling. No neurological or vascular deficits were observed. Radiographic findings revealed a nondisplaced fracture of the distal radius that did not extend into the radioulnar or radiocarpal interarticular area (Figure 1).

A splint, incorporating synthetic cotton, was applied to stabilize the distal radius metaphyseal fracture. The patient received comprehensive explanations regarding all necessary information, potential complications, and detailed recommendations. These included guidance on elevation, intermittent ice application, the use of paracetamol as needed, and scheduled check-ups at the orthopedics and traumatology outpatient clinic. The patient was instructed to monitor neurovascular deficits closely.

**Figure 2:**
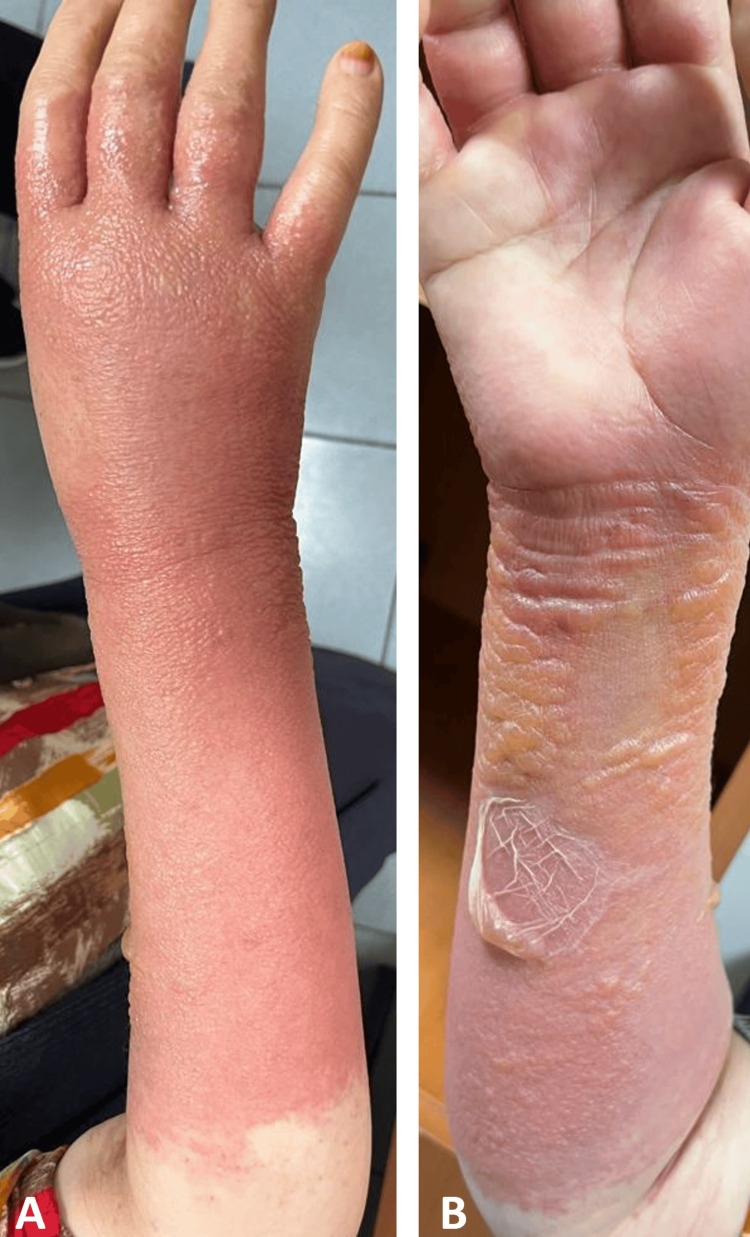
Skin lesions after removing the splint application The posterior side of the forearm (2A) and anterior side of the forearm (2B) views after removing the splint displayed itchy, reddened, dense blisters compatible with the splint line

One day later, the patient reported severe itching and burning in the right arm. By the morning of the second day, causing a significant escalation in the complaint of burning, the splint was removed from the emergency room. A skin lesion characterized by intense itching, redness, and dense blisters emerged along the splint line, extending from the proximal interphalangeal joint area to the forearm elbow region (Figure 2).

**Figure 3:**
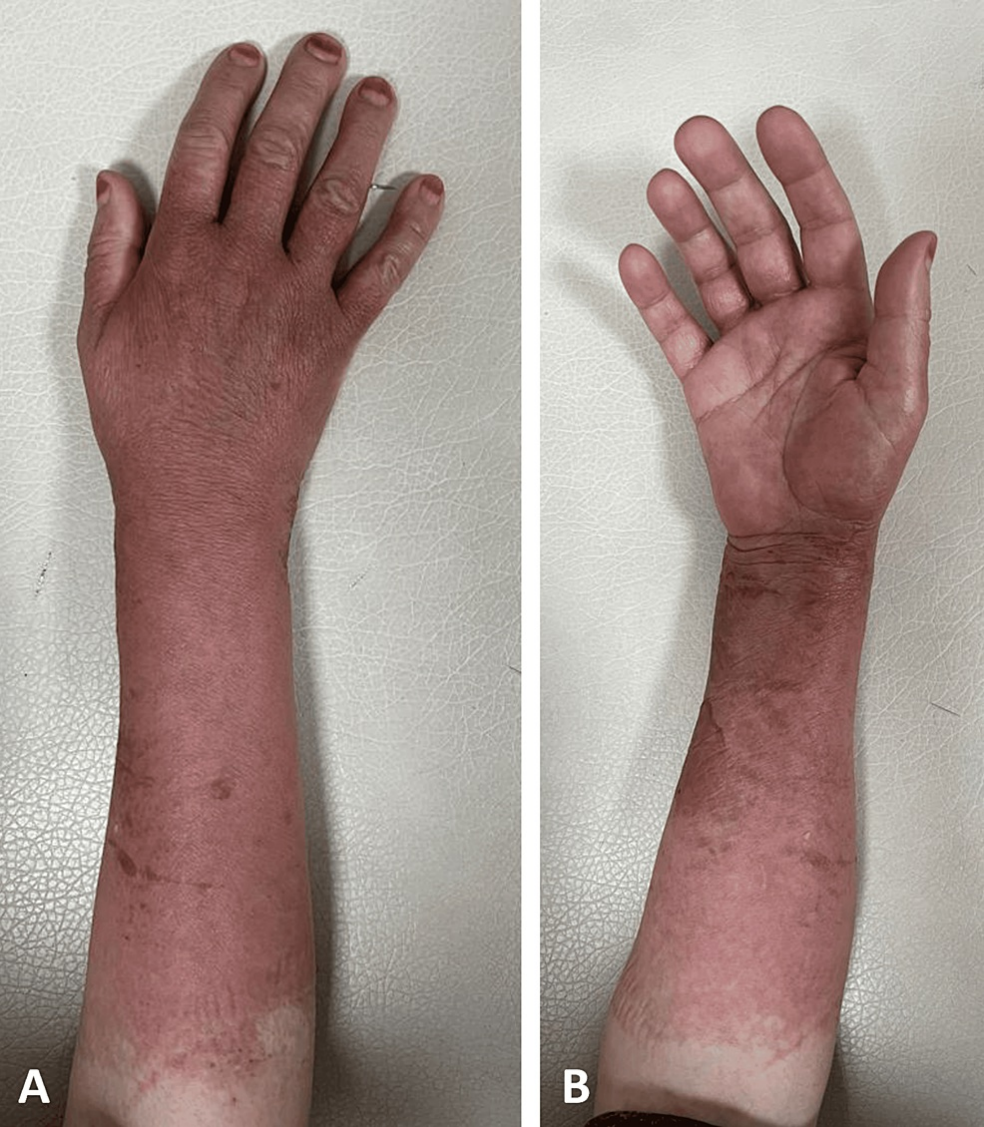
Three days after treatment check for ACD The posterior side of the forearm (3A) and anterior side of the forearm (3B) views after treatment, demonstrating regression of the skin lesions

After the removal of the splint, the patient's fracture treatment was continued with a shoulder-arm sling until the lesions healed. The patient was subsequently referred to the dermatology department, where a diagnosis of ACD due to undercast cotton padding was established by the dermatologist. Treatment commenced with a daily dosage of 40 mg methylprednisolone for five days, supplemented by local antihistamine cream application. After three days of treatment, the patient underwent a follow-up examination, revealing a regression of the skin lesions. Additionally, the patient disclosed a history of never using menstrual pads due to skin irritation (Figure 3).

**Figure 4:**
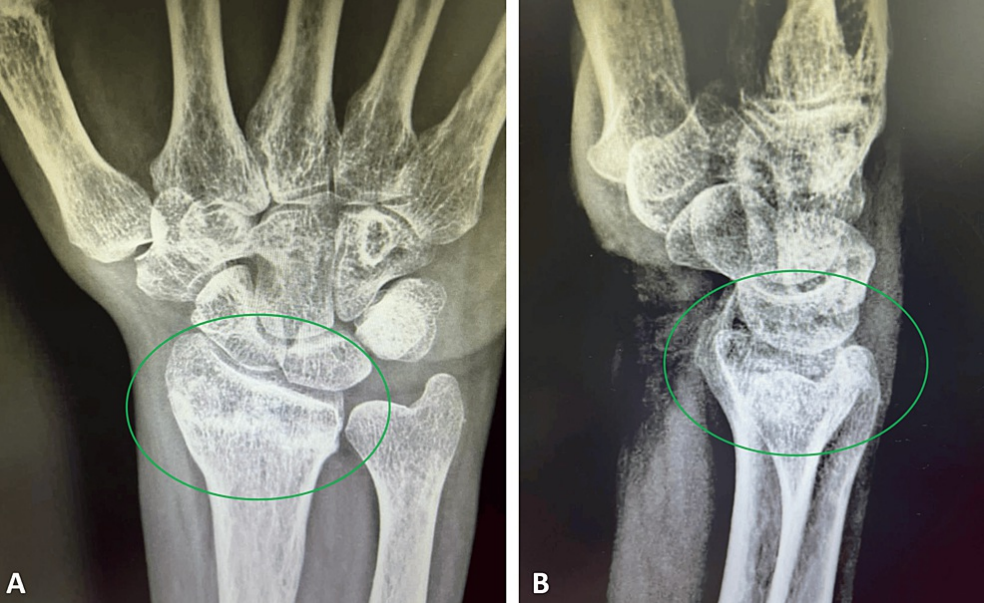
Fifth week of the follow-up Wrist anteroposterior (4A) and lateral (4B) views of the patient after five weeks of treatment indicated the healing of the distal radius fracture with callus formation

**Figure 5:**
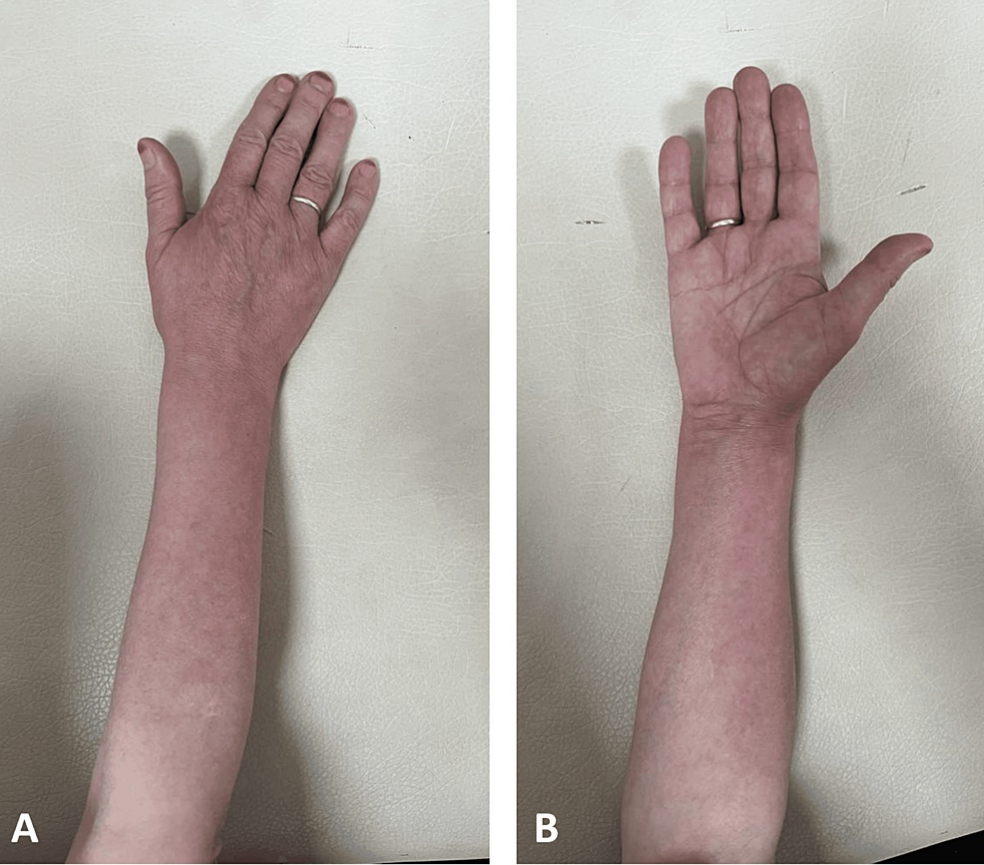
Fifth week of the follow-up The posterior side of the forearm (5A) and anterior side of the forearm (5B) views during the fifth-week check revealed only minimal remaining skin color changes

At the approximately fifth-week follow-up, the distal radial fracture had healed (Figure 4), exhibiting a full, painless range of motion with only minimal residual skin color changes (Figure 5). The patient was reassured that the remaining skin color alterations would resolve in the upcoming weeks.

## Discussion

Splint application typically represents the primary treatment modality for certain unstable fractures or dislocations. Padded fiberglass or plaster stands as the most commonly employed materials for splinting. Despite its prevalence, splint application is frequently executed inappropriately or inadequately [1]. Common challenges associated with splinting include acute musculoskeletal injuries leading to a loss of fracture reduction, skin irritation or disruption of its integrity, and joint stiffness. Exothermic reactions resulting from splinting materials like casting can potentially lead to thermal injuries [1,5,6]. Furthermore, rare but significant complications involve compartment syndrome and neurovascular deficits [1].

ACD induced by undercast cotton padding is an infrequent occurrence in orthopedic clinical practice and is scarcely documented in orthopedic literature [7-10]. Following an ACD episode, it is imperative to differentiate skin lesions from cellulitis and other dermatological conditions. Cellulitis, a common acute bacterial skin infection, manifests with poorly demarcated redness, swelling, warmth, and pain in the infected skin region without the presence of an abscess or purulent discharge [11]. Distinct clinical findings and skin lesions must be discerned from those of ACD. Patients with cellulitis typically exhibit elevated body temperature and increased acute-phase reactants. In contrast, a lack of fever or leukocytosis, coupled with a history of allergic reactions post the use of sanitary pads and a positive response to corticosteroid treatments, leans toward the diagnosis of ACD. Plaster of Paris, containing benzalkonium chloride, a quaternary ammonium compound, has been identified as a potential cause of ACD [7]. Given the widespread use of benzalkonium chloride in daily products such as disinfectants, antiseptics, cosmetics, deodorants, mouthwashes, toothpaste, creams, and ophthalmic preparations [12], avoiding these products safeguards against subsequent allergic episodes.

In contemporary orthopedics and traumatology practice, synthetic casts, molded from fiberglass, are preferred to prevent contact dermatitis triggered by plaster of Paris or undercast cotton padding. Alternatively, utilizing a stockinette on the affected area followed by wrapping in synthetic cotton padding ensures that skin-irritating materials do not directly contact the skin, thus minimizing the risk of sensitization. Fiberglass casts offer advantages such as increased porosity compared to plaster casts, reducing the vulnerability of the skin underneath the cast to irritation [13]. Our patient experienced ACD resulting from the cotton used in the splint. If a plaster cast had been used after a stockinette on the affected area for our patient, perhaps an allergic reaction would not have occurred. This is because using a stockinette on the affected area followed by wrapping in synthetic cotton padding helps prevent direct contact between skin-irritating materials and the skin, thereby reducing the risk of sensitization.

## Conclusions

We have observed favorable outcomes in a 56-year-old female patient who developed ACD following the application of a splint for a distal radial fracture. One of our key takeaways is the critical importance of scheduling a follow-up appointment with the patient one day after the application of the cast or even the splint. Not only neurovascular issues, but, as seen here, skin problems can also arise after casts and even splints. Additionally, patients should be educated about warning signs, including skin irritation, neurovascular deficits (such as assessing capillary refill), and symptoms indicative of compartment syndrome. This ensures the timely identification of any significant pathological developments. Healthcare practitioners must not overlook inquiring about patients' histories of allergic skin reactions. This proactive approach aids in preventing ACD through early intervention and preventive measures.
